# mTORC2 regulates hierarchical micro/nano topography‐induced osteogenic differentiation via promoting cell adhesion and cytoskeletal polymerization

**DOI:** 10.1111/jcmm.16672

**Published:** 2021-06-10

**Authors:** Qian Gao, Yuying Hou, Zhe Li, Jinyang Hu, Dawei Huo, Huimin Zheng, Junjiang Zhang, Xiaoyu Yao, Rui Gao, Xudong Wu, Lei Sui

**Affiliations:** ^1^ Department of Prosthodontics Tianjin Medical University School and Hospital of Stomatology Tianjin China; ^2^ Tianjin Key Laboratory of Medical Epigenetics Department of Cell Biology 2011 Collaborative Innovation Center of Tianjin for Medical Epigenetics Tianjin Medical University Tianjin China; ^3^ Department of Neurosurgery Tongji Hospital Tongji Medical College Huazhong University of Science and Technology Wuhan China; ^4^ International Education College Tianjin University of Traditional Chinese Medicine Tianjin China

**Keywords:** cell adhesion, cytoskeleton, hierarchical micro/nano structure, mTORC2, osteogenic differentiation

## Abstract

Surface topography acts as an irreplaceable role in the long‐term success of intraosseous implants. In this study, we prepared the hierarchical micro/nano topography using selective laser melting combined with alkali heat treatment (SLM‐AHT) and explored the underlying mechanism of SLM‐AHT surface‐elicited osteogenesis. Our results show that cells cultured on SLM‐AHT surface possess the largest number of mature FAs and exhibit a cytoskeleton reorganization compared with control groups. SLM‐AHT surface could also significantly upregulate the expression of the cell adhesion‐related molecule p‐FAK, the osteogenic differentiation‐related molecules RUNX2 and OCN as well as the mTORC2 signalling pathway key molecule Rictor. Notably, after the knocked‐down of Rictor, there were no longer significant differences in the gene expression levels of the cell adhesion‐related molecules and osteogenic differentiation‐related molecules among the three titanium surfaces, and the cells on SLM‐AHT surface failed to trigger cytoskeleton reorganization. In conclusion, the results suggest that mTORC2 can regulate the hierarchical micro/nano topography‐mediated osteogenesis via cell adhesion and cytoskeletal reorganization.

## 1 INTRODUCTION

Implant surface topography can regulate cell behaviour and ultimately be involved in cell fate decisions.^1^, ^2^, ^3^ For dental implants, the clinical widely used grit‐blasted and acid‐etched (SLA) titanium surface with single micro‐scale topography which has been proved much better in osteointegration than smooth (S) titanium surface, partly due to the facilitating role of the micro‐scale structure in bone locking and implant initial stability.^4^, ^5^, ^6^ However, it was reported that the single micro‐scale feature might inhibit cell attachment and proliferation.^6^, ^7^ To achieve long‐term success of the intraosseous implants, it is necessary to alleviate the inhibitory effects of the single micro‐scale topography. Recently, the hierarchical micro/nano topography has attracted extensive attention since the nano‐scale feature can increase the adsorption of proteins and subsequently enhance cell attachment.^8^, ^9^ Notably, mimicking the natural bone structure which consists of micro‐scale collagen fibers and nano‐scale hydroxyapatite, the hierarchical micro/nano topography titanium surface provides a better microenvironment than that with signal micro‐scale topography for cell‐surface interaction.^10^, ^11^, ^12^, ^13^, ^14^, ^15^ In our previous study, we have revealed that the hierarchical micro/nano topography was superior to the SLA titanium surface in improving the osteogenesis.^16^, ^17^ However, the elaborate regulation process of the cell‐surface interaction and the underlying mechanism have remained to be elucidated.

Cells perceive the implant surface through various of mechanosensors. It is widely known that cell adhesion and actin cytoskeleton have a central role in sensing and transmitting extracellular stimuli based on the connection between cell membranes and nuclears mechanically. Biochemically, cell adhesion is mediated by the integrin (at the nano‐scale).^18^ The interaction and gathering of integrin result in the assembly of several intracellular ankyrins (talin, vinculin, etc) to induce the formation of mature focal adhesion (FA) (at micro‐scale), which connects the material surface and the cytoskeleton to propagate the biochemical signalling.^19^ Among the diverse adhesion‐related signalling pathways, focal adhesion kinase (FAK) was considered as an important one regulating the actin cytoskeleton organization‐related signallings, including Cdc42, Rac and ROCK, as well as participating in cell fate decisions.^20^ Concomitantly, linked to the adhesion‐related molecules, actin cytoskeleton also acts as a critical modulator generating intracellular tension which contributes to the regulation of cell phenotype. Overall, compelling evidence supports a critical role of the cell adhesion and actin cytoskeleton in the hierarchal micro/nano topography‐elicited osteogenesis.^12^, ^21^, ^22^ Our former works have indeed shown that the micro/nano topography could direct cell fate via promoting cell adhesion,^16^ polymerization of cytoskeleton, and the regulation of chromatin modifications.^17^ However, relatively little has been known about the molecular mechanism how topography regulates cell adhesion and cytoskeleton up to now. Herein, we sought to observe the underlying mechanism of the hierarchical microgroove/nanopore topography we fabricated in regulating the cell adhesion, actin cytoskeleton and finally osteogenic differentiation.

Mammalian target of rapamycin (mTOR) is an evolutionarily conserved serine/threonine protein kinase which could interact with different proteins and form two functionally distinct complexes termed mTORC1 and mTORC2. mTORC1 involves Raptor as a unique adaptor protein rather than Rictor in mTORC2. mTOCR1 can sense various environmental conditions, like insulin and serum, coordinating multiple cell processes from catabolism and anabolism of protein and lipid to autophagy.^23^, ^24^, ^25^ By contrast, the main function of mTORC2 is to phosphorylate AGC subfamily of kinases, such as AKT and PKCα, which regulate cell proliferation, survival and actin cytoskeleton,^25^, ^26^ while the function and underlying mechanism of mTORC2 are still in the exploratory stage.^27^ In recent years, growing evidence has implicated that mTORC2 plays a critical role in bone homeostasis.^28^, ^29^, ^30^ Rictor knock‐down in mature osteoblast and BMSCs resulted in impaired osteogenic differentiation in vitro and compromised bone formation in vivo.^31^, ^32^ In the process of osteoblast differentiation, mTORC2 was activated as the downstream of canonical Wnt signalling pathway.^33^, ^34^ In turn, osteogenic gene RUNX2 could directly bind to the promoter of mTOR and activate the mTORC2/AKT signalling pathway.^35^, ^36^ The phosphorylation of Akt‐Ser473, the best characterized substrate of mTORC2, has been demonstrated to be necessary for osteogenesis.^37^ Given that Rictor has been found to regulate cytoskeleton through PKCα initially,^38^, ^39^ and overall proteome analyses have shown that the function of mTORC2 was highly associated with cell adhesion in cancer cells,^40^ we supposed that mTORC2 might be responsible for the topographical cues‐induced osteogenic differentiation, through regulating cell adhesion and cytoskeletal polymerization.

In the presented study, we fabricated the titanium surface with hierarchal microgroove/nanopore topography by using the selective laser melting (SLM) technique combined with alkali heat treatment (AHT). We hypothesized that, during cell reading hierarchal micro/nano topography, mTORC2 was activated to enhance cell adhesion and cytoskeletal polymerization, which in turn promoted osteogenesis. To verify this, we first proved that cell adhesion and mTORC2 signalling pathway could be activated by the hierarchal micro/nano topography. Moreover, Rictor stable knock‐down MC3T3‐E1 cells were used to confirm the role and underlying mechanism of mTORC2 in topographical cues‐induced osteogenic differentiation. Our results demonstrated that mTORC2 was essential in this process. In the absence of mTORC2 signalling, topographical cues‐induced signalling transduction based on the cell adhesion and the actin cytoskeletal polymerization will be blocked and consequently impair the osteogenesis.

## 2 MATERIALS AND METHODS

### 2.1 Titanium surfaces preparation and topography observation

Three groups of specimens (Ti‐6Al‐4V) were used in the following experiments, including the smooth surface obtained by machining and polishing (S), the micro‐scale surface fabricated by sandblast, large grit and acid etching (SLA) and the hierarchical micro/nano surface constructed by the selective laser melting combined with alkali heat treatment (SLM‐AHT). S group was prepared by polishing with sandpaper from 240 to 2000 grits sequentially. SLA group was fabricated as is previously described.^17^ SLM‐AHT group was prepared by selective laser melting (SLM). The key parameters of the SLM system were set as follows: laser spot size of 0.1, wavelength of 1054 nm, scanning speed of 7 m‐s and continuous power of 200 W. Then, the specimens were treated by 5 mol/L NaOH at 100℃ for 2 hours. Next, the specimens were heated in muffle furnace from 0 ℃ to 600 ℃ (5℃/min). The resultant titanium specimens were cleaned ultrasonically in acetone, absolute ethanol and double‐distilled water (ddH2O) sequentially for 15 min and sterilized at 120℃/2 h. The specimens were sterilized with ultraviolet light for at least 30 minutes before use.

A Field‐emission scanning electron microscope (FE‐SEM SUPRA 55 SAPPHIRE, Germany) was employed to observe the titanium surface topography. To obtain micro‐scale and nano‐scale surface morphology, both of the low and high magnification images were observed in each group.

### 2.2 Cell culture and osteogenic induction

MC3T3‐E1 cells were purchased from American Type Culture Collection (ATCC), and cultured in fresh DMEM (Gibco) with 1% penicillin/streptomycin and 10% FBS (Gibco) in 5% CO_2_ atmosphere at 37℃.

For osteogenic induction (OI), the culture medium was changed on the next day with the medium consisted of DMEM, 10% FBS, 1% penicillin/streptomycin, 50 μg/ml ascorbic acid (Sigma), 5 mmol/L β‐glycerophosphate (Sigma) and 10 nmol/L dexamethasone (Sigma).

### 2.3 Construction of Rictor knock‐down cell lines

To explore the role of mTORC2 in the hierarchical micro/nano topography‐induced osteogenesis, we designed two short hairpin RNAs (shRNAs) to knock down Rictor in MC3T3‐E1 cells. Scramble shRNA was employed as a control group. Rictor‐shRNA oligos (as shown in Table 1) were purchased from GENEWIZ (China), and scramble‐shRNA plasmid was preserved in our laboratory. Briefly, shRNA oligos were annealed and ligated into digested pLKO.1 vector, and the correctly identified sequence was transfected into 293T cells using PAX8 (packaging) and VSVG (enveloping) plasmid. Virus supernatants were harvested 48 hours later to infect MC3T3‐E1 cells at 70% confluence. After 48 hours infection, puromycin was added to selected positive cells. RT‐qPCR and Western blot were used to examine the Rictor knock‐down efficiency in the method detailed in the corresponding sector.

**TABLE 1:** Rictor shRNA sequences

Gene	shRNA sequences (5′‐3′)
Rictor‐shRNA1	F:CCGGCGAGACTTTGTCTGTCTAATTCTCGAGAATTAGACAGACAAAGTCTCGTTTTTG R:AATTCAAAAACGAGACTTTGTCTGTCTAATTCTCGAGAATTAGACAGACAAAGTCTCG
Rictor‐shRNA2	F:CCGGGCCATCTGAATAACTTCACTACTCGAGTAGTGAAGTTATTCAGATGGCTTTTTG R:AATTCAAAAAGCCATCTGAATAACTTCACTACTCGAGTAGTGAAGTTATTCAGATGGC

### 2.4 MTS

To evaluate the cells’ proliferation, scramble cells and Rictor knock‐down cells were seeded at a density of 1 × 10^3^ cells/well. After 24 hours, 2 and 4 days culturing, the culture medium was replaced by empty DMEM, MTS and PMS (100:20:1) and then cells were cultured at 37℃ for 2 hours. Finally, the absorbance at 490 nm wavelength was detected and OD value was calculated.

### 2.5 Immunoprecipitation

To investigate the interaction of vinculin and Rictor, immunoprecipitation was performed. Wild‐type cells cultured on the three surfaces were collected and lysed on ice for 30 minutes, sonicated and centrifugated, and 3% supernatant was collected as input. Vinculin antibody (CST) was added to the incubation at 4℃ overnight, followed by 3‐hour incubation with Protein A/G beads (Smart‐Lifesciences). Immunoprecipitates were washed three times and resuspended when buffer is loaded for SDS‐PAGE analysis.

### 2.6 Immunofluorescence

To visualize FA formation, the state of the actin cytoskeleton and the subcellular localization of Rictor, wild‐type cells and Rictor knock‐down cells were seeded at a density of 1 × 10^3^ cell / well on three different surfaces for 24 hours. To detect the positive stain state of the osteogenic differentiation‐related molecule RUNX2, wild‐type cells, scramble cells and Rictor knock‐down cells were seeded at a density of 1 × 10^4^ cell / well on three different surfaces for 3 days and 7 days. The cells mentioned above were fixed with 4% paraformaldehyde for 10 minutes, permeabilized with 0.05% Triton X‐100 (Sigma‐Aldrich) for 5 minutes and blocked with 5% BSA for 30 minutes, followed by incubation with primary antibodies of RUNX2 (CST) and Rictor (CST) overnight, F‐actin (1:200, Molecular Probes) for 30 minutes and secondary antibody (1:200, Invitrogen) for 1 hour. DAPI was employed to stain cell nuclei. Finally, the moderate mounting media (changjia) was added on microscope slide (changjia), then the specimen was putted on the microscope slide carefully to prepared for subsequent confocal image observation.

### 2.7 Confocal image observation and image analysis

To detect the effect of the hierarchical micro/nano topography on mature FA formation, wild‐type cells, scramble cells and Rictor knock‐down cells were seeded at a density of 1 × 10^3^ cell/well on three different surfaces for 24 hours. Vinculin was employed to represent FA, and was stained according to the protocol of immunofluorescence described above. Confocal images were observed by using Zeiss Axio Imager M2 Optical Microscope (Carl Zeiss, Germany). The mature FA was defined as in the previous study we have published.^16^ Briefly, Image J software was employed to calculate the area of vinculin stain. The area which was greater than 3.14 µm^2^ was acted as mature FA.

### 2.8 RT‐qPCR

Wild‐type cells were seeded at a density of 1 × 10^4^ cell/well on three titanium surfaces for 24 hours to detect the gene expression levels of the adhesion‐related gene vinculin and mTORC2 signalling molecule Rictor. Wild‐type cells, scramble cells and Rictor knock‐down cells were cultured at a density of 1 × 10^4^ cell/well on three titanium surfaces for 3 days and 7 days to detect the gene expression levels of osteogenic differentiation‐related molecule RUNX2, OCN and mTORC2 signal pathway molecule Rictor. Total RNA was extracted by Trizol (Invitrogen), and the equivalent mRNA of each group was reversely transcribed into cDNA. LightCycler® 480 SYBR Green I Master (Roche) was employed to perform RT‐qPCR analyses. All data were normalized to Rpo. The primer sequences are listed in Table 2.

**TABLE 2:** RT‐qPCR Primer sequences

Gene	Primer sequences (5′‐3′)
RP0	F: TTCATTGTGGGAGCAGAC R: CAGCAGTTTCTCCAGAGC
vinculin	F: ACCTGCAGACCAAAACCAAC R: CTTACCGACTCCACGGTCAT
RUNX2	F: ATCACTGACGTGCCCAGGCGTA R: AGGGCCCAGTTCTGAAGCACCT
OCN	F: AGTCTGACAAAGCCTTCA R: AAGCAGGGTTAAGCTCACA
Rictor	F: GCTGCGCTATCTCATCCAAGA R: GGTTCTGAAGTGCTAGTTCAC

### 2.9 Western blot

Wild‐type cells, scramble cells and Rictor knock‐down cells were seeded at a density of 1 × 10^4^ cell / well on the different surfaces for 24 hours to detect the protein expression levels of adhesion‐related molecules FAK, p‐FAK and vinculin, for 3 days and 7 days to detect the protein expression levels of osteogenic differentiation‐related molecule RUNX2 and mTORC2 signalling pathway molecules mTOR, Rictor, AKT and p‐AKT. The cells mentioned above were collected and lysed in RIPA on ice, then centrifuged and denatured. The proteins from each sample were loaded to run SDS polyacrylamide gel electrophoresis, and then transferred to a PVDF membrane, followed by blocking the unspecific protein binding sites in 5% BSA. Then, the membrane was incubated with primary antibody overnight at 4℃ and proper secondary antibodies for 1 hour at room temperature. Primary antibodies used are listed as follows: FAK (CST), p‐FAK (CST), vinculin (CST), RUNX2 (CST), Rictor (CST), mTOR (CST), Rictor (CST), AKT (CST) and p‐AKT (CST).

### 2.10 Statistical analysis

To ensure the validity of data, all experiments were repeated at least three times. Experimental data were tested for homogeneity followed by one‐way ANOVA analysis. All error bars represent mean ±standard deviation (SD) (n = 3). *P* <.05 was considered significant (∗*P* <.05, ∗∗*P* <.01, ∗∗∗*P* <.005, ∗∗∗∗*P* <.001).

## 3 RESULTS

### 3.1 Surface topography

**FIGURE 1:**
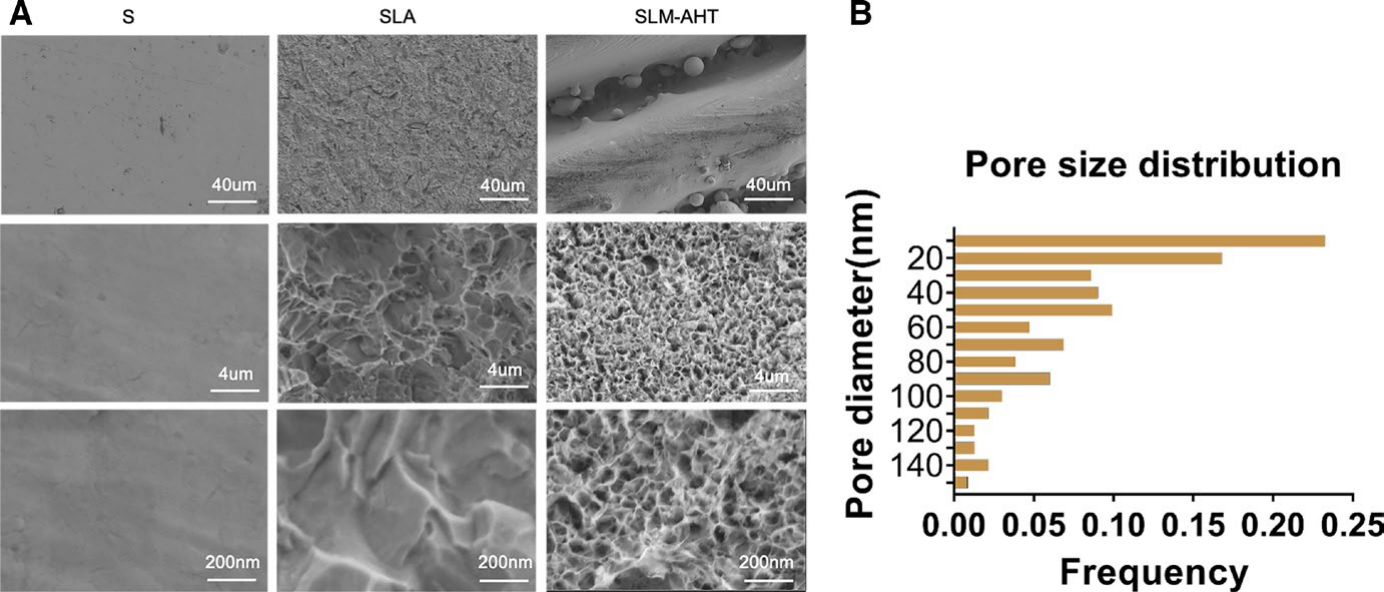
Surface observation of S, SLA and SLM‐AHT titanium specimen. A, FE‐SEM observation of S, SLA and SLM‐AHT group topography at 200×, 20 000× and 50 000× magnification. B, The size distribution of the nanopores on SLM‐AHT surface

As the FE‐SEM images shown in Figure 1A, SLM‐AHT titanium surface presented the hierarchical microgroove/nanopore topography with micron‐sized groove in an average of 30‐40 μm and the nano‐sized mesh‐like pore with a diameter of approximately 10–100 nm. Most of the nanostructure on SLM‐AHT surface was about 50 nm in diameter (Figure 1B). S titanium surface possessed no apparent micro or nano structures. SLA titanium surface exhibited typical micro‐scale structure without nano‐scale feature.

### 3.2 The effect of hierarchical micro/nano topography on cell adhesion, actin cytoskeleton and eventually cell osteogenesis

**FIGURE 2:**
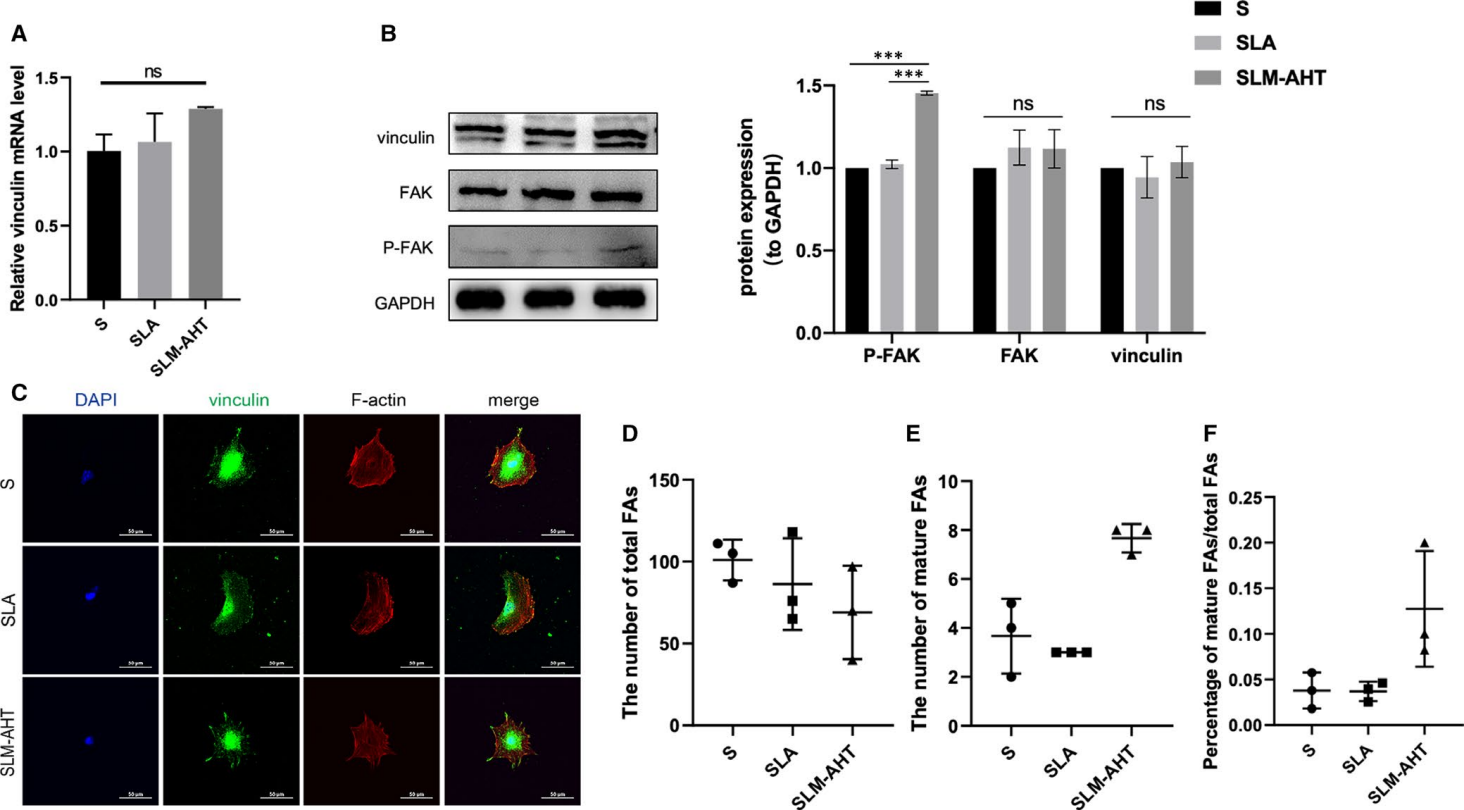
The effect of different titanium surfaces on cell adhesion and cytoskeletal polymerization after 24 h of culture. A, The gene expression level of vinculin. B, The protein expression levels of vinculin, FAK and P‐FAK. C, Immunofluorescence staining (red, F‐actin; green, vinculin; blue, DAPI). D, Total FAs number. E, Mature FAs number. F, The percentage of mature FAs/total FAs

After 24 hours of culture, there was no significant difference in the expression levels of FAK and vinculin among the three titanium surfaces. However, MC3T3‐E1 cells on the SLM‐AHT surface displayed a remarkably enhanced protein expression level of p‐FAK (Figure 2A,B), demonstrating that the SLM‐AHT surface could activate adhesion‐related FAK signalling pathway. Furthermore, as shown in Figure 2C‐F, cells on SLM‐AHT surface exhibited fewer FAs in total but more mature FAs than they did in the other two groups, suggesting that SLM‐AHT surface could promote mature FAs formation.

As shown in Figure 2C, cells cultured on SLM‐AHT surface showed a typical polygonal, elongated morphology, and the actin fibres were arranged in an orderly way with higher intensity. In contrast, cells on control groups were round in shape, and the cytoskeleton was in a disorderly state with lower intensity, indicating SLM‐AHT surface could trigger the polymerization of the cytoskeleton.

**FIGURE 3:**
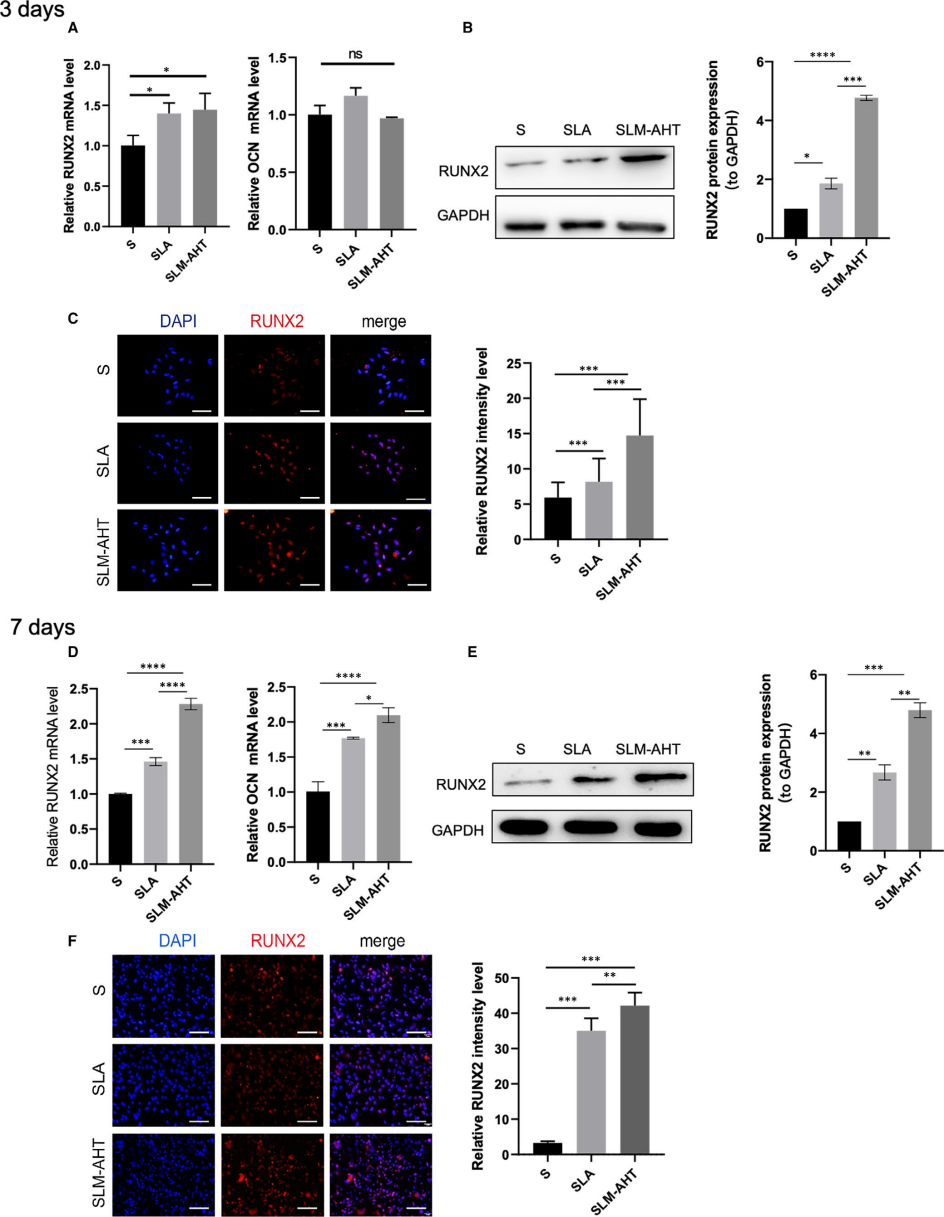
Hierarchical micro/nano topography could promote osteogenesis. A, The gene expression levels of RUNX2 and OCN of cells cultured on three titanium surfaces for 3 days. B, The protein expression level of RUNX2 of cells cultured for 3 days. C, (left panel) Immunofluorescence staining for RUNX2 of cells cultured for 3 days (red, RUNX2; blue, DAPI; scale bar: 200 µm), (right panel) fluorescence staining intensity analysis. D, The gene expression levels of RUNX2 and OCN of cells cultured for 7 days. E, The protein expression level of RUNX2 of cells cultured for 7 days. F, (left panel) Immunofluorescence staining for RUNX2 of cells cultured for 7 days (red, RUNX2; blue, DAPI; scale bar: 200 µm), (right panel) fluorescence staining intensity analysis

After 3 days of culture, the gene expression level of RUNX2 was slightly enhanced, while the protein expression level of RUNX2 was significantly enhanced on SLM‐AHT surface (Figure 3A,B). After 7 days of culture, a considerable increased expression of RUNX2 were detected on SLM‐AHT surface in comparison with control groups (Figure 3D,E). Consistently, the strongest RUNX2 positive stain was observed in the cells cultured on SLM‐AHT surface (Figure 3C,F). Meanwhile, the gene expression level of late‐stage osteogenic differentiation marker OCN was notably increased after 7 days instead of 3 days of culture on SLM‐AHT surface (Figure 3A,D). Collectively, it could be inferred that SLM‐AHT surface has greater potential in promoting cell osteogenic differentiation than the single micro‐scale surface.

### 3.3 The role of mTORC2 in topographical cues‐induced cell osteogenic differentiation

**FIGURE 4:**
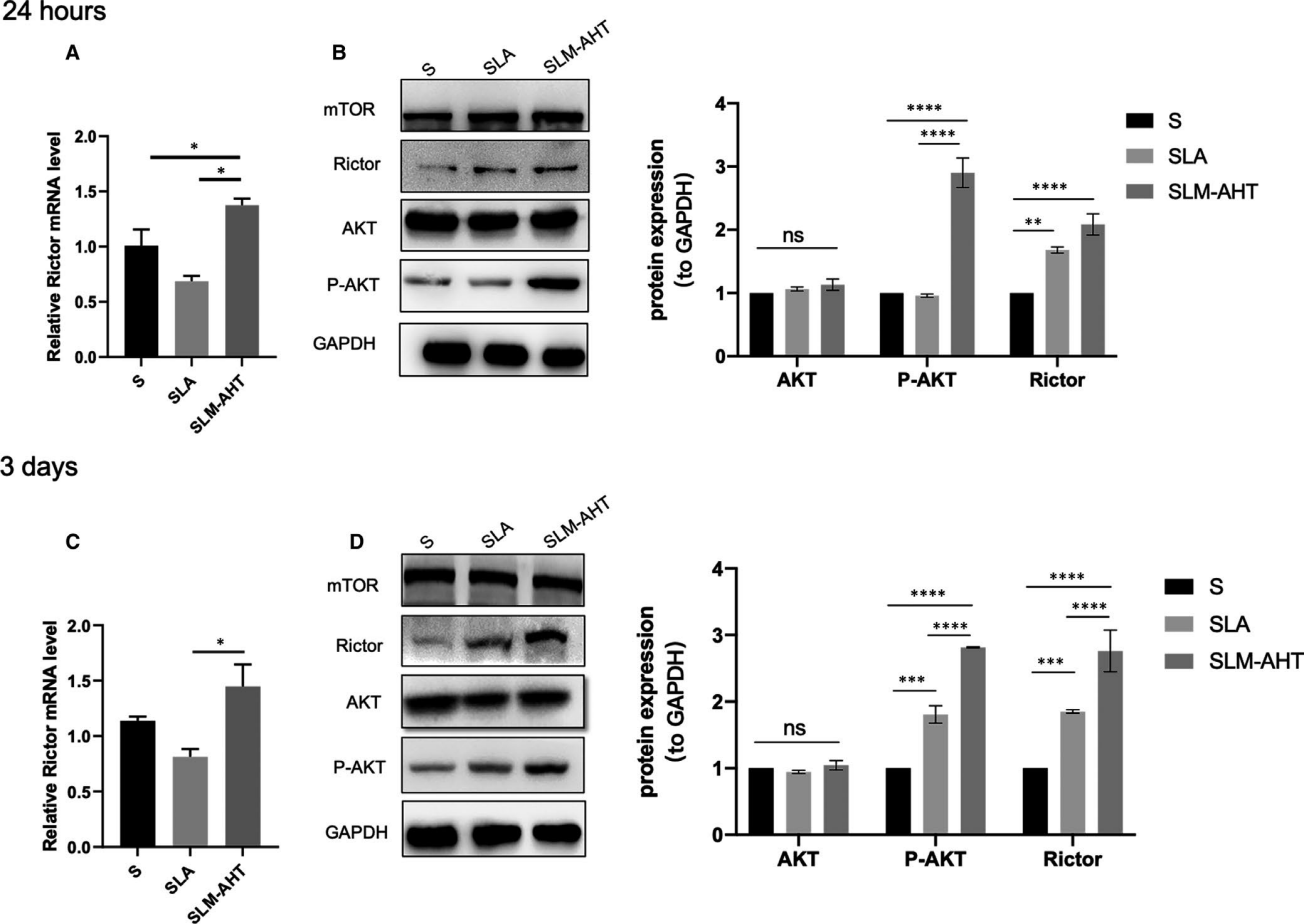
Hierarchical micro/nano topography could facilitate the activation mTORC2 signalling pathway. A, The gene expression level of Rictor in cells cultured for 24 h. B, The proteins expression levels of mTOR, Rictor, AKT and P‐AKT in cells cultured for 24 h. C, The gene expression level of Rictor in cells cultured for 3 days. D, The proteins expression levels of mTOR, Rictor, AKT and P‐AKT in cells cultured for 3 days

As shown in Figure 4, SLM‐AHT group could significantly enhance the gene expression level of Rictor, the protein expression levels of Rictor and p‐AKT, indicating that hierarchical micro/nano topography could facilitate the activation of the mTORC2 signalling pathway.

**FIGURE 5:**
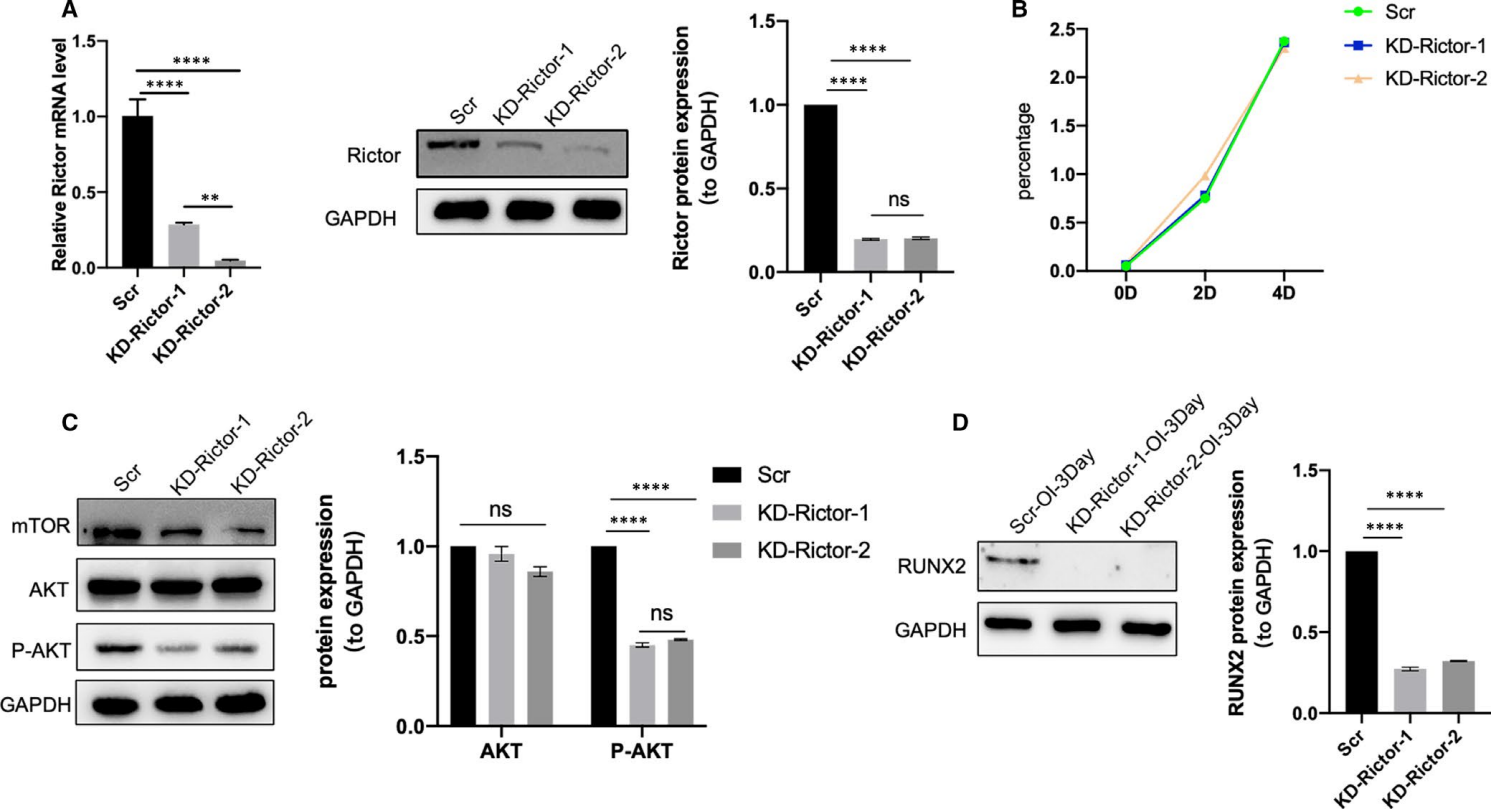
mTORC2 is necessary for osteogenesis. A, The gene and protein expression levels of Rictor in scramble and Rictor knock‐down cells. B, MTS analysis of scramble and Rictor knock‐down cells. C, The proteins expression levels of mTOR, AKT and P‐AKT in scramble and Rictor knock‐down cells. D, The protein expression level of RUNX2 in scramble and Rictor knock‐down cells for 3 days osteoinduction

**FIGURE 6:**
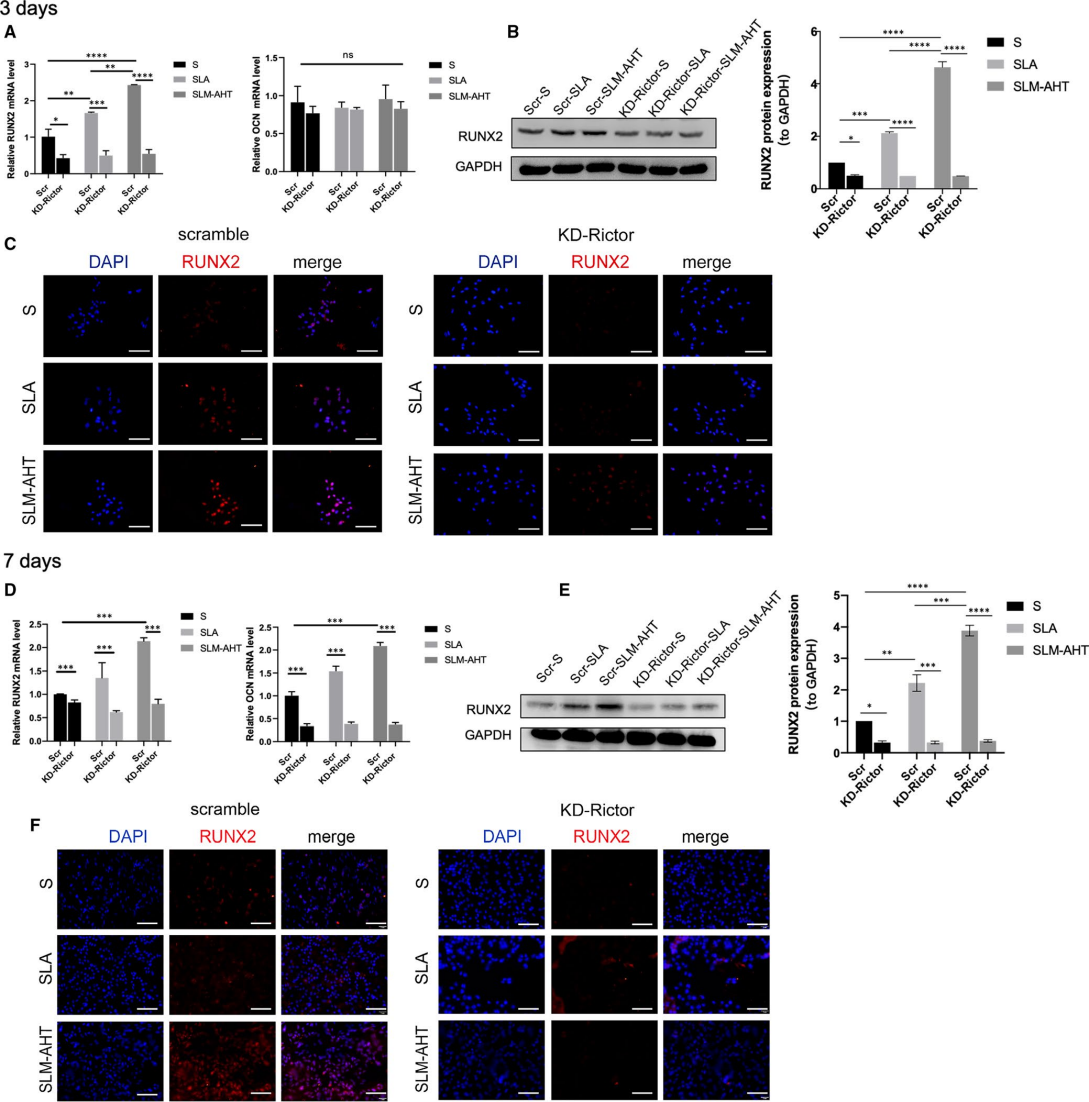
mTORC2 is essential for hierarchical micro/nano topography‐induced osteogenesis. A, The gene expression levels of RUNX2 and OCN of scramble and Rictor knock‐down cells cultured for 3 days. B, The protein expression level of RUNX2 of scramble and Rictor knock‐down cells cultured for 3 days. C, Immunofluorescence staining assays for RUNX2 of scramble and Rictor knock‐down cells cultured for 3 days (blue, DAPI; red, RUNX2; scale bar: 200 µm). D, The gene expression levels of RUNX2 and OCN in scramble and Rictor knock‐down cells cultured on different group for 7 days. E, The protein expression level of RUNX2 in scramble and Rictor knock‐down cells cultured for 7 days. F, Immunofluorescence staining assays for RUNX2 in scramble and Rictor knock‐down cells cultured for 7 days (blue, DAPI; red, RUNX2; scale bar: 200 µm)

To explore the functional role of mTORC2 in hierarchical micro/nano topography‐mediated osteogenesis, we constructed Rictor stable knock‐down MC3T3‐E1 cell lines by short‐hairpin RNA. The Rictor knock‐down was effective (Figure 5A), and did not affect cells proliferation (Figure 5B). After Rictor knock‐down, the protein expression level of p‐AKT was decreased (Figure 5C), indicating that mTORC2/AKT signalling pathway was blocked effectively. After 3 days of osteoinduction, the protein expression level of RUNX2 in Rictor knock‐down cells was significantly downregulated (Figure 5D), demonstrating that the downregulation of mTORC2 could impair osteogenesis. Then, the expression levels of RUNX2, OCN in Rictor knock‐down cells and scramble cells cultured on the three titanium surfaces were detected. The results were shown in Figure 6. After 3 days and 7 days of culture, the scramble cells on the SLM‐AHT surface displayed tremendously upregulated expression levels of RUNX2 and OCN (Figure 6A,B,D,E), whereas Rictor knock‐down cells on the SLM‐AHT surface displayed a similar expression levels of those factors compared with the other two surfaces. Furthermore, immunofluorescence results showed that the downregulation of mTORC2 resulted in similar RUNX2 positive stain among the three titanium surfaces, while scramble cells displayed a considerably enhanced RUNX2 positive stain on the SLM‐AHT surface compared with the control surfaces (Figure 6C,F). In all, it could be inferred that mTORC2 was essential for SLM‐AHT surface‐induced osteogenesis.

### 3.4 The relationship between mTORC2 activation and hierarchical micro/nano topography‐induced cell adhesion and cytoskeletal polymerization

**FIGURE 7:**
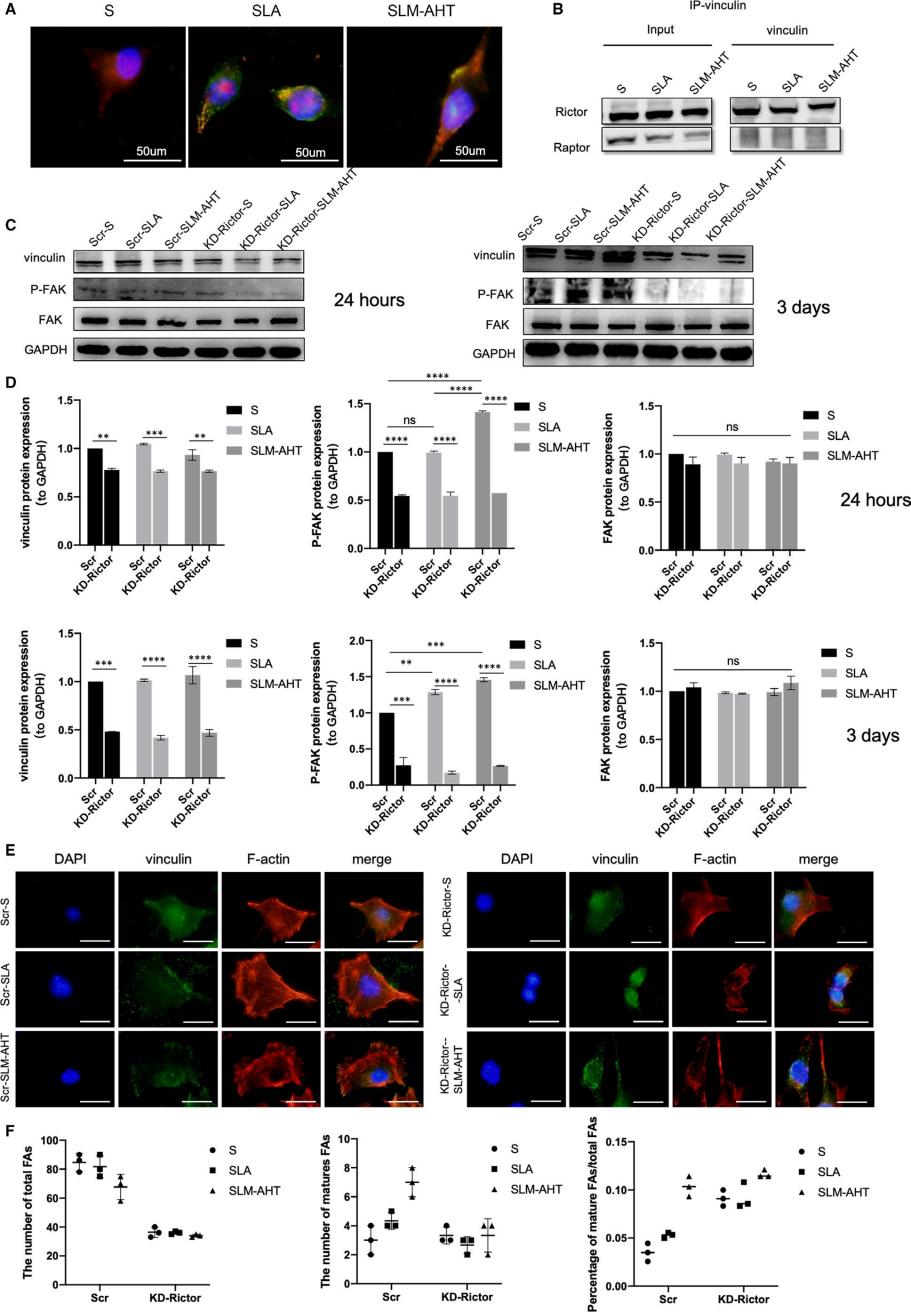
mTORC2 is required for hierarchical micro/nano topography‐induced cell adhesion and cytoskeletal polymerization. A, Immunofluorescence staining assay for the subcellular localization of Rictor and vinculin in cells cultured on different titanium surfaces for 24 h (red, Rictor; green, vinculin; blue, DAPI). B, Immunoprecipitation of vinculin in cells cultured for 24 h. C, The proteins expression levels of vinculin, FAK and P‐FAK of cells cultured for 24 h and 3 days. D, The quantitative analysis of (C). E, Immunofluorescence staining assays for the state of focal adhesion and F‐actin in scramble and Rictor knock‐down cells cultured for 24 h (blue, DAPI; green, vinculin; red, F‐actin; scale bar: 50 um). F, The number of total FAs, mature FAs and the percentage of mature FAs/total FAs of scramble and Rictor knock‐down cells cultured for 24 h

To further unravel the molecular mechanism of mTORC2 in hierarchical micro/nano topography‐mediated osteogenesis, immunofluorescence was employed to visualize the subcellular localization of Rictor. Rictor was colocalized with vinculin, and mainly located on the cell membrane on the SLM‐AHT surface in a punctuated pattern, whereas mainly distributed in cytoplasm on the S and SLA surfaces (Figure 7A), suggesting that the SLM‐AHT surface could induce cell membrane localization of Rictor. Then, immunoprecipitation results demonstrated that there was a direct interaction between Rictor and vinculin on titanium surfaces, while Raptor was not (Figure 7B), indicating that vinculin was specifically bound to mTORC2 rather than mTORC1. Based on those results, we then hypothesized that mTORC2 participated in the regulation of SLM‐AHT surface‐elicited cell adhesion. Therefore, we detected the protein expression level of adhesion‐related molecules after Rictor knock‐down. As shown in Figure 7C,D, Rictor knock‐down could downregulate the protein expression levels of vinculin and p‐FAK. Additionally, immunofluorescence was employed to observe the state of focal adhesion in Rictor knock‐down cells on the three titanium surfaces. As shown in Figure 7F, the deletion of Rictor led to a decreased in the number of mature FAs and the total number of FAs. These data indicated that mTORC2 was essential for the SLM‐AHT surface‐induced cell adhesion.

Meanwhile, the state of the actin cytoskeleton was observed after Rictor knock‐down. As expected, after Rictor knock‐down, the SLM‐AHT surface failed to promote the polymerization of the actin cytoskeleton (Figure 7E), demonstrating that mTORC2 was required for SLM‐AHT surface‐induced cytoskeletal polymerization.

## 4 DISCUSSION

To date, various factors have been proved to be capable of influencing the osseointegration of intraosseous implants. Among those, surface topography has been regarded as an indispensable parameter conducing to the success of dental implants. The differences in surface topography from micro‐scale to nano‐scale might produce different effects on osteogenesis. Emerging evidence has revealed that the hierarchical micro/nano topography has a great potential in promoting osteogenesis compared with the single micro or nano topography since it possesses the mixed advantages that micro‐scale structure could reinforce the interlocking of the bone with the implant^41^ while nano‐scale structure could increase protein adsorption, cell adhesion and ultimately osseointegration.^8^, ^42^ However, the underlying mechanism that surface topography manipulates cell fate still requires further investigation. In the current study, we utilized SLM to fabricate microgroove titanium surface, on which AHT was employed to create nanopore features. The resultant specimens were used to explore the effect of the SLM‐AHT surface on cell adhesion, actin cytoskeleton and eventually osteogenesis.

Adhesion of cells to implant surface was considered as the very beginning of the osseointegration.^18^, ^43^, ^44^ In the present study, we fully proved that the SLM‐AHT surface could promote the process of cell adhesion. At the molecular level, our results showed protein expression level of p‐FAK was remarkably enhanced in cells cultured on the SLM‐AHT surface. Interestingly, there was no significant difference in the expression level of vinculin among the three titanium surfaces, probably because the activation of vinculin mainly depended on its construction instead of its increased total mRNA and protein expression level.^45^, ^46^ At micro‐scale, since the mature of FA could indicate the true state of cell adhesion,^16^ we calculated the number of mature FAs. Our results demonstrated that the cells cultured on SLM‐AHT surface exhibited an increased number of mature FAs compared with the other two groups. Moreover, adhesion‐driven changes in cell shape could rapidly promote polymerization of cytoskeleton. Notably, fitting the size of microscale topography, actin filaments allowed the cell to align along with the micro features to generate intercellular force, which could lead to larger adhesion formation and promote osteogenesis.^47^, ^48^ Our results confirmed that the SLM‐AHT surface indeed could trigger polymerization of actin cytoskeleton. The expression levels of osteogenic differentiation molecules RUNX2 and OCN were enhanced significantly, indicating that the cells on SLM‐AHT surface performed better osteogenic differentiation than those on S and SLA. Together, the above‐mentioned theories and results suggested that the SLM‐AHT surface could enhance cell adhesion and polymerization of the cytoskeleton, which in turn account for why the SLM‐AHT surface was conducive to cell osteogenesis. Nevertheless, little has been known about the mechanism of cell adhesion and polymerization of the cytoskeleton triggered by hierarchical micro/nano topography. Thus, in the follow‐up experiments, we investigated the underlying mechanism.

Compelling evidence has indicated that mTORC2 plays a crucial role in regulating bone homeostasis including both bone formation and absorption.^49^, ^50^, ^51^ Given the evidence that mTORC2 was sensitive to mechanical cues and was essential in osteogenesis,^32^ we have conducted the following experiments to systematically explore the effect of mTORC2 in SLM‐AHT surface‐mediated osteogenesis. Gene expression level of Rictor and protein expression levels of Rictor and p‐AKT were considerably enhanced in SLM‐AHT group, indicating that mTORC2/AKT signalling pathway could be activated by hierarchical micro/nano topography. Furthermore, the disabled Rictor experiment showed that there was no longer a significant difference in osteogenic differentiation among S, SLA and SLM‐AHT surfaces, suggesting that mTORC2 played an irreplaceable role in SLM‐AHT surface‐induced osteogenesis.

We further determined the regulation mechanism involved in mTORC2 in topographical cues‐induced osteogenesis. Since the function of protein is strongly correlated with its subcellular location,^52^, ^53^ we performed immunofluorescence to observe the subcellular localization of Rictor. Interestingly, we observed the existence of colocalization of vinculin and Rictor on the cell membrane. Immunoprecipitation was further employed to confirm the relationship between Rictor and vinculin, and our result indicated that the interaction between the two proteins indeed occurred. Thus, we hypothesized that mTORC2 was responsible for SLM‐AHT surface‐induced cell adhesion from the perspective of FAs formation. To verify this hypothesis, we stained vinculin to observe the effect of Rictor on FAs formation on different titanium surfaces. And our results showed that, after Rictor knock‐down, vinculin failed to exhibit a punctate pattern on the cell membrane. Besides, the vinculin expression level was downregulated after Rictor knock‐down, demonstrating that mTORC2 was essential for hierarchical micro/nano topography‐induced FA’s formation. Moreover, previous studies have reported that responding to mechanical cues, mTORC2 could be regulated by the FAK signalling pathway.^32^, ^54^ Notably, embedded in FA, p‐FAK displayed an elongated and aligned pattern on the nanoscale topography.^55^ Proceeding from this angle, we sought to explore whether mTORC2 could mediate hierarchical micro/nano topography‐induced adhesion‐related signalling pathway transduction. Our results showed that, after Rictor knock‐down, the protein expression level of p‐FAK decreased, suggesting that mTORC2 could mediate hierarchical micro/nano topography‐induced adhesion‐related signalling pathway transduction. Collectively, in this study, we observed that Rictor knock‐down cells on the SLM‐AHT surface showed a decrease in cell adhesion through the FA formation and adhesion‐related signalling transduction. This is the first to disclose that mTORC2 could regulate the cell adhesion triggered by hierarchical micro/nano topography.

And as we have known, actin cytoskeleton was linked to adhesion‐related molecules, which allowed the transduction of mechanical cues to regulate the downstream signalling pathways.^56^ Based on previous studies that have reported that mTORC2 could regulate cytoskeletal polymerization through Rho family GTPases in response to soluble factors,^27^, ^39^ and the finding from this study that mTORC2 was involved in SLM‐AHT surface‐induced cell adhesion and eventually osteogenesis,^57^ we suppose that mTORC2 could also act as the upstream molecule of the actin cytoskeleton in response to hierarchical micro/nano topography. As expected, our results showed that after the knock‐down of Rictor, SLM‐AHT surface failed to promote the polymerization of the actin cytoskeleton, indicating that hierarchical micro/nano topography acts as a mechanical cue resulting in mTORC2 activation to regulate actin cytoskeletal polymerization. Accordingly, our results demonstrated that the mTORC2 signalling pathway could enable and augment topographical cues which provided a new area for hierarchical micro/nano topography‐mediated cell fate decisions. However, the upstream regulation of mTORC2 remains a key unresolved question in the process of cell‐reading hierarchical micro/nano topography. mTORC2 could be activated by a variety of biochemical signallings, such as WNT/LRP5 ^34^ and Hedgehog,^58^ involved in the regulation of osteogenesis.^59^ Therefore, mTORC2‐involved biochemical signalling pathway in SLM‐AHT surface‐mediated osteogenesis still awaits further investigation.

In conclusion, mTORC2 activation in response to hierarchical microgroove/nanopore topography leads to enhancement of cell adhesion and polymerization of the cytoskeleton, which allows for an amplification of topographical cues orchestrating cell osteogenic differentiation. It is considered that there was an interaction between mechanical and biochemical signalling pathways, as well an interplay between the intrinsic and the extrinsic mechanical environment. Further experiments are needed to explore the more detailed molecular mechanisms both in vivo and in vitro.

## CONFLICT OF INTEREST

No potential conflicts of interest.

## AUTHOR CONTRIBUTION

**Qian Gao:** Conceptualization (lead); Data curation (equal); Investigation (lead); Writing‐original draft (equal); Writing‐review & editing (equal). **Yuying Hou:** Investigation (equal); Software (equal). **Zhe Li:** Investigation (supporting); Methodology (equal). **Jinyang Hu:** Data curation (equal); Methodology (equal); Software (equal); Visualization (equal). **Dawei Huo:** Methodology (equal); Software (equal). **Huimin Zheng:** Methodology (supporting). **Junjiang Zhang:** Conceptualization (supporting); Methodology (equal). **Xiaoyu Yao:** Methodology (equal); Software (equal). **Rui Gao:** Writing‐review & editing (equal). **Xudong Wu:** Conceptualization (equal); Supervision (equal). **Lei Sui:** Conceptualization (equal); Funding acquisition (lead); Supervision (equal); Writing‐original draft (equal); Writing‐review & editing (equal).

## Data Availability

Data are available on request from the authors.
